# Willingness to use long-acting injectable PrEP among HIV-negative/unknown men who have sex with men in mainland China: A cross-sectional online survey

**DOI:** 10.1371/journal.pone.0293297

**Published:** 2023-10-19

**Authors:** Jiaqi Fu, Zhenwei Dai, Hao Wang, Mingyu Si, Xu Chen, Yijin Wu, Weijun Xiao, Yiman Huang, Fei Yu, Guodong Mi, Xiaoyou Su

**Affiliations:** 1 School of Population Medicine and Public Health, Chinese Academy of Medical Sciences & Peking Union Medical College, Beijing, China; 2 Danlan Public Welfare, Beijing, China; University of New South Wales, AUSTRALIA

## Abstract

**Background:**

Men who have sex with men (MSM) are at high risk of HIV acquisition. Long-acting injectable-pre-exposure prophylaxis (LAI-PrEP), requiring less frequent dosing, is being studied as an alternative method to daily oral HIV PrEP. With the addition of this potential new prevention method, it expands the scope for a wider user choice and is expected to increase the acceptability and uptake of HIV prevention measures. The aim of our study was to explore the willingness to use LAI-PrEP and associated influential factors.

**Methods:**

Participants were recruited from December 2020 to March 2021 through banner advertisements on web- and mobile app-based platforms on Blued, a large gay Chinese social media platform. MSM in our cross-sectional study was HIV-negative and currently lived in mainland China. Participants were asked about their willingness to use LAI-PrEP and reasons why they might be or not be willing to use LAI-PrEP. Multivariable logistic regression was used to analyze the factors associated with the willingness to use LAI-PrEP.

**Results:**

In total, 969 participants met the inclusion criteria and finished the survey. Nearly twenty percent (19.5%) of participants had never tested for HIV; 66.8% of MSM had multiple male partners; and 51.6% of MSM engaged in condomless sex with their partner. About three-fifths (66.3%) of MSM were aware of PrEP, and only 3.9% of MSM had used PrEP before. The willingness to use LAI-PrEP among MSM was 74.0% (95% CI: 71.4%-76.6%). MSM with higher education levels were less likely to show a willingness to use LAI-PrEP (AOR = 0.56, 95%CI: 0.38–0.84). Participants who had a history of HIV test (AOR = 1.68, 95%CI: 1.11–2.55), were willing to use daily oral PrEP (AOR = 10.64, 95%CI:7.43–15.21), had multiple male sexual partners (AOR = 1.33, 95%CI:0.93–1.90), who used rush popper(AOR = 1.49, 95%CI:1.05–2.13), and who were aware of PEP (AOR = 1.66, 95%CI: 1.02–2.70) were more likely to show willingness to use LAI-PrEP.

**Conclusions:**

In our study, MSM had quite high awareness but low uptake of PrEP. As LAI-PrEP is expected to be approved for use in China in the future, our study of MSM highlights the need for key population-focused education programs about PrEP and healthy sexual behavior. This study also provides some evidence for LAI-PrEP use among the Chinese MSM population in the future.

## Introduction

Men who have sex with men (MSM) are at high risk of HIV acquisition [1]. The Joint United Nations Programme on HIV/AIDS (UNAIDS) reported that there were roughly 1.5 million new HIV infections worldwide in 2021, with MSM accounting for 17% of those infections [2]. In China, 23.3% of new cases of HIV infections were attributed to homosexual transmission in 2020 [3]. The overall prevalence of HIV among MSM in China was 5.7% from 2001 to 2018 and has a trend of continuous rise [4]. At present, the main preventive measures against HIV include increasing condom use, increasing HIV testing frequency, and reducing the number of sexual partners [5–7]. However, the new infections globally have not been controlled effectively [8]. It is necessary to explore another effective HIV prevention strategy in addition to condom use, regular testing, and reducing the number of partners among MSM. At present, pre-exposure prophylaxis (PrEP) has proven effective at preventing HIV infection and has been recommended by the World Health Organization (WHO) to prevent HIV transmission among HIV-negative MSM in 2015 [9–11]. There is a wide range of acceptability for PrEP among MSM on a global scale, from as little as 5.7% to 100% [12]. Previous studies demonstrated that although Chinese MSM had low awareness (11%-13%) of PrEP, their willingness to use PrEP was high (64%-92%) after learning about its safety and efficacy of PrEP [13–15]. Truvada is the most popular HIV PrEP formula due to its high effectiveness when used correctly [16,17]. This PrEP is a single tablet containing tenofovir disoproxil fumarate and emtricitabine [17]. On August 11, 2020, Truvada (emtricitabine and tenofovir disoproxil fumarate tablets, emtricitabine 200 mg/tenofovir disoproxil fumarate 300 mg, FTC/TDF) was approved by China’s National Medical Products Administration (NMPA) as PrEP in people at high risk of HIV infection [18]. As of right now, MSM at risk for HIV infection can obtain PrEP online or at infectious disease hospitals in mainland China.

Daily oral PrEP, the main form of PrEP currently available, could reduce incident HIV infection by 44% among MSM [9]. The current price of domestically produced oral PrEP in China ranges from 300 to 500 RMB per month. In addition, the price of imported Truvada is 1980 RMB per month. A study investigating the cost-effectiveness of oral PrEP and expanded antiretroviral therapy (ART) for preventing HIV infections among MSM in China found that 20% or 50% PrEP combined with 90% ART would be cost-effective, with incremental cost-effectiveness ratios (ICERs) of 25,417 and 47,243, respectively. Additionally, reducing the annual cost of oral PrEP by 64% would make it highly cost-effective for 50% PrEP combined with 90% ART [19]. However, the increase in PrEP use in China has been slow due to several factors, including the lack of a national strategy plan and guidelines for its use in preventing HIV transmission, the absence of innovative and applicable models for PrEP service delivery, and limited community engagement in PrEP initiatives [20,21]. Hence, most MSM in China would be transferred to the infectious department of a designated hospital for treatment after being infected [22]. Therefore, the promotion and acceptance of PrEP in China warrant attention. The effectiveness of PrEP is highly dependent on PrEP adherence [23,24]. Poor adherence to daily PrEP would impair the efficacy of daily oral PrEP and remains a concern among MSM [25,26]. At present, a novel modality of PrEP, long-acting injectable- (LAI-) PrEP that requires less frequent dosing is being studied as an alternative method to daily oral PrEP [27–29]. The World Health Organization (WHO) has recently issued new guidelines advocating the use of LAI-PrEP for the prevention of HIV globally since it is a safe and highly effective prevention method for individuals at substantial risk of HIV infection, and China is expected to implement trials of LAI-PrEP in MSM in the future [30,31]. Phase 2b/3 trials of LAI-PrEP have also demonstrated its efficacy. For example, the HPTN 083 study found that long-acting injectable cabotegravir (CAB-LA) was more effective than daily oral tenofovir disoproxil fumarate–emtricitabine (TDF–FTC) in preventing HIV infection among cisgender MSM (MSM) and transgender women who have sex with men [32]. On December 20, 2021, the U.S. Food and Drug Administration granted approval for Apretude® (cabotegravir extended-release injectable suspension) to be used as a PrEP medication, making it the first long-acting injectable option for the primary prevention of HIV. LAI-PrEP may be able to circumvent some of the adherence issues associated with daily oral PrEP, such as remembering to take medicine daily, pill fatigue over time, or unintended disclosure of PrEP use to partners [33–36]. At the same time, as a different form from daily oral PrEP, LAI-PrEP could help improve the coverage of PrEP among MSM due to its convenience and longer protection duration [28]. Gorden et al. found a high willingness (74%) among daily oral PrEP users to use LAI-PrEP due to the inconvenience of daily oral PrEP [37]. In a study by Meyers et al., 80% of young MSM in New York City expressed interest in using LAI-PrEP, and Parsons et al. found that 46% of gay and bisexual men preferred LAI-PrEP compared to oral PrEP [38,39]. Therefore, we suppose that Chinese MSM will also have a high willingness to use LAI-PrEP when it is available in China. It is of great significance for public health professionals to identify which populations prefer to use LAI-PrEP for strategy to promote the use of LAI-PrEP among MSM in the future. Compared with high-income countries, only a few studies conducted in local health service centers in China investigated the willingness to use LAI-PrEP [1,40–42].

As a new HIV prevention method, LAI-PrEP expands the scope for a wider user choice and is expected to increase the acceptability and uptake of PrEP [43]. As one of the steps of the PrEP cascade, knowing the willingness to use PrEP among MSM is crucial to PrEP use [44]. This research might offer insight into the level of interest in LAI-PrEP among MSM, and can inform strategies for scaling up LAI-PrEP to target populations and provide direct evidence for policy-making service and planning in terms of PrEP implementation among MSM in China [45].

## Methods

### Participants and procedures

This was a cross-sectional, online, and national study among mainland Chinese MSM. Participants were recruited from December 2020 to March 2021 by means of the Blued app through private messages with the slogan in Chinese: “Peking Union Medical College and Light Blue Public Welfare invite you to participate in HIV/AIDS prevention research and contribute to a better future for the gay community!”. And the data was also collected by Blued app, which is a large gay Chinese social media platform. The inclusion criteria were as follows: (1) born biologically male; (2) aged 18 and above; (3) self-reported anal intercourse with at least one man in the last six months; (4) self-reported HIV-negative or unknown; and (5) currently live in mainland China. Exclusion criteria: people who were unable to complete the questionnaire. Each user can fill in the questionnaire only once according to the UID of each user. According to the sample size calculation formula of the cross-sectional study and the previous research in Guangzhou about the willingness of PrEP (79.5%), a sample size of 696 produced a two-sided 95% confidence interval with a width equal to 0.06[46]. Electronic informed consent was obtained from all participants through an online informed consent form before the questionnaire. Eligible participants completed an online survey in Chinese. To ensure confidentiality, participants entered their data directly into a computer-based questionnaire. All participants were distinguished by the number, which could not identify individual participants during and after collection. Ethics approval was obtained from the ethics committee of Danlan Beijing Media Limited on May 20, 2020 (Number: DLIRB202005-01).

### Measurements

Our original questionnaire was independently developed in Chinese with reference to the previous literature by our research team. A panel of specialists in epidemiology, psychology, and behavioral science was further invited to review and evaluate the face validity of the questionnaire, and to make the final modification suggestion. Finally, according to the suggestions of specialists, our questionnaire contained 63 items and was structured. The questionnaire has also been validated by a pilot study conducted by our research team to make sure the items were understandable. Sociodemographic information was collected including age, ethnicity, marital status, work or study status, education level, personal monthly income, and sexual orientation. Items assessing sexual risk behaviors included multiple male sexual partners in the past 6 months (individuals who had more than one male partner were classified as having multiple male sexual partners), HIV positive status among sexual partners in the past 6 months, type of sexual partners (regular/casual sexual partners)(casual sexual partners refer to those participants had sex with but were not in the committed, intimate relationship)[47], sex with sex workers in the past 6 months (yes/no), sex with females in the past 6 months (yes/no), usage of rush popper in the past 6 months (yes/no) (rush popper is a smooth muscle relaxant, which is usually used to relax the anal sphincter and to help MSM achieve rapid sexual arousal in a short period of time) [48], and consistent condom use (namely, always using a condom during the past sex) in the last 6 months (yes/no) [49,50]. We also asked participants whether they had tested for HIV in the past. The situation of awareness and usage of post-exposure prophylaxis (PEP, one of the biomedical interventions for HIV infection) was asked in similar ways. The awareness and usage of post-exposure prophylaxis (PEP, one of the biomedical interventions for HIV infection) were measured by the questions “Have you ever heard of PEP?” and “Have you ever used PEP?”.

HIV knowledge was measured by an HIV knowledge questionnaire (HIV-KQ-18), which has been validated among various population [51,52]. This scale consists of 18 judgment questions, with the answer options for questions on HIV knowledge being “true”, “false” or “don’t know”. 1 point is awarded for correct responses, and 0 for incorrect responses or responses not known. The higher the total score, the more HIV prevention knowledge was understood.

Participants were asked whether they had ever heard of any type of PrEP and used PrEP before the survey. The participants were told that LAI-PrEP was a long-acting injection for HIV prevention that was administered every two months [53]. The question of willingness to use LAI-PrEP was asked as “If there is a long-acting PrEP drug that is injected every two months, would you be willing to use it?” The willingness to use LAI-PrEP was asked on a 5-point Likert scale (1 = strongly willing to use LAI-PrEP, 2 = willing to use LAI-PrEP, 3 = uncertain, 4 = unwilling to use LAI-PrEP, 5 = definitely unwilling to use LAI-PrEP). Participants were classified into the “willing to use LAI-PrEP” group when they endorsed responses 1 or 2 and into the “unwilling to use LAI-PrEP” group when they endorsed responses 3, 4, or 5. For MSM who were or were not willing to use LAI-PrEP, a further question was asked about the reasons. And we allowed participants to give multiple responses. The measurement of willingness to use daily oral PrEP was similar to the measurement of willingness to use LAI-PrEP.

### Data analysis

Descriptive analyses were used to summarize the socio-demographic characteristics and sexual behaviors of the sample. We computed descriptive statistics, including frequencies and percentages for categorical variables, and means and standard deviations for continuous variables. The primary outcome was willingness to use LAI-PrEP which was measured as a proportion with its confidence interval. Logistic regression models were used to identify correlates of the willingness to use LAI-PrEP. Univariable logistic regression was used to identify associations between the willingness to use LAI-PrEP and the variables. All factors with p<0.1 in univariable logistic regression or considered relevant in previous studies were then included in the multivariable logistic regression models. Odds ratios (OR) and adjusted odds ratios (AOR) were reported with 95% confidence intervals (95%CI) in logistic regression models. All analyses were conducted in IBM SPSS Statistics (version 21, SPSS Inc., Chicago, IL, USA), and two-tailed p<0.05 was considered statistically significant.

## Results

### Sociodemographic characteristics and sexual behaviors

Of the 969 MSM who self-reported HIV-negative or unknown status, 72.5% were below 36 years old. Most participants were of Han ethnicity (88.4%), were unmarried (76.0%), had a full/part-time job (69.9%), and had obtained a bachelor’s degree or above (73.2%). Over half earned CNY 3000–9999 per month (53.4%). Nearly three-quarters (74.9%) of the participants identified themselves as gay. Most MSM had previously tested for HIV (80.5%) in their lifetime. In the past 6 months, about two-thirds had more than one sexual partner (66.8%), and 34.8% only had sex with a regular partner. About ten percent (11.4%) of participants reported ever having sex with at least one partner who was HIV positive. Approximately half (51.6%) of the participants reported consistent condom use in the past 6 months. Rush poppers were used by 43% of MSM (43.2%) in the previous 6 months (Table 1).

**Table 1:** Sociodemographic characteristics and sexual behaviors of HIV-negative/unknown MSM in China (N = 969).

Variables		Frequency		Percentage
**Age (years)**				
18–25		332		34.3%
26–35		370		38.2%
≥36		267		27.6%
**Ethnicity**				
Han		857		88.4%
Others		112		11.6%
**Marital status**				
Unmarried		736		76.0%
Married		136		14.0%
Divorced/Widowed		97		10.0%
**Work or study status**				
Full/part-time job		677		69.9%
Student		219		22.6%
Unemployed/Retired		73		7.5%
**Education**				
High school or below		260		26.8%
University or above		709		73.2%
**Monthly income (CNY)**				
<3000		295		30.4%
3000–9999		517		53.4%
≥10000		157		16.2%
**Sexual orientation**				
Gay		726		74.9%
Other		243		25.1%
**Ever had HIV tested**				
No		189		19.5%
Yes		780		80.5%
**Had multiple male sexual partners**				
No		322		33.2%
Yes		647		66.8%
**Had HIV-positive sexual partners**				
No		859		88.6%
Yes		110		11.4%
**Type of sexual partners**				
regular sexual partners		337		34.8%
casual sexual partners		632		65.2%
**Had sex with sex workers**				
No		916		94.5%
Yes		53		5.5%
**Had sex with female**				
No		856		88.3%
Yes		113		11.7%
**Consistent condom use**				
No		469		48.4%
Yes		500		51.6%
**Ever used rush popper**				
No		550		56.8%
Yes		419		43.2%
**HIV knowledge**			13.76±3.17	

### Awareness of PrEP and willingness to use LAI-PrEP

Nearly two-thirds (66.3%) of MSM reported ever hearing about any type of PrEP, however only less than 4 percent (3.9%) had ever used PrEP. The willingness to use LAI-PrEP was 74.0% (95%CI: 71.4%-76.6%). More than sixty percent (64.1%) of the participants heard about PEP, and less than 10% (8.4%) of MSM ever used PEP (Table 2).

**Table 2:** Awareness and willingness for PrEP among HIV-negative/unknown MSM in China (N = 969).

Variables	Frequency	Percentage
**Heard of PrEP**		
No	327	33.7%
Yes	642	66.3%
**Ever used PrEP**		
No	931	96.1%
Yes	38	3.9%
**Willingness to use daily oral PrEP**		
No	227	23.4%
Yes	742	76.6%
**Willingness to use LAI-PrEP**		
No	252	26.0%
Yes	717	74.0%
**Heard of PEP**		
No	348	35.9%
Yes	621	64.1%
**Ever used PEP**		
No	888	91.6%
Yes	81	8.4%

### Univariable factors associated with willingness to use LAI-PrEP

In the univariable logistic analysis, participants who were divorced or widowed were more willing to use LAI-PrEP compared with participants who were unmarried (OR = 1.76, 95%CI:1.02–3.04). Participants with higher education levels had lower willingness to use LAI-PrEP (OR = 0.74, 95%CI:0.53–0.94). MSM who had ever taken an HIV test (OR = 1.82, 95%CI:1.30–2.56) and reported two or more sexual partners in the past 6 months (OR = 1.57, 95%CI:1.17–2.11) were more likely to be willing to use LAI-PrEP. The history of using rush poppers in the past 6 months was significantly and positively associated with the willingness to use LAI-PrEP (OR = 1.84, 95%CI:1.36–2.49). Participants who had ever heard of PrEP (OR = 1.67, 95%CI:1.24–2.24) were more likely to choose LAI-PrEP. Men who showed a willingness to use daily oral PrEP had a higher likelihood of choosing LAI-PrEP relative to those unwilling to use daily oral PrEP (OR = 9.60, 95%CI:6.86–13.44). Participants who had ever heard of PEP (OR = 1.71, 95%CI:1.27–2.29) and had ever used PEP (OR = 21.75, 95%CI:0.97–3.18, p = 0.065) were more likely to use LAI-PrEP (Table 3).

**Table 3:** Univariable factors associated with willingness to use LAI-PrEP (N = 969).

Variables	Willingness to use LAI-PrEP			
	Yes(n(%))/Mean ± Standard deviations		OR	p
**Age (years)**				
18–25	234(70.5%)	1		
26–35	280(75.7%)	1.30(0.93–1.82)		0.121
≥36	203(76.0%)	1.33(0.92–1.92)		0.129
**Ethnicity**				
Han	633(73.9%)	1		
Others	84(75.0%)	1.06(0.67–1.67)		0.796
**Marital status**				
Unmarried	536(72.8%)	1		
Married	101(74.3%)	1.08(0.71–1.63)		0.728
Divorced/Widowed	80(82.5%)	1.76(1.02–3.04)		0.044
**Work or study status**				
Full/part-time job	505(74.5%)	1		
Student	157(71.7%)	0.86(0.61–1.21)		0.395
Unemployed/Retired	55(75.3%)	1.04(0.60–1.82)		0.889
**Education**				
High school or below	203(78.1%)	1		
University or above	514(72.5%)	0.74(0.53–0.94)		0.008
**Monthly income (CNY)**				
<3000	216(73.2%)	1		
3000–9999	386(74.7%)	1.08(0.78–1.49)		0.652
≥10000	115(73.2%)	1.00(0.65–1.55)		0.995
**Sexual orientation**				
Gay	540(74.4%)	1		
Other	177(72.8%)	0.92(0.67–1.28)		0.636
**Ever had HIV tested**				
No	121(64.0%)	1		
Yes	596(76.4%)	1.82(1.30–2.56)		0.001
**HIV knowledge**	13.84±3.082	1.03(0.99–1.08)		0.185
**Multiple male sexual partners**				
No	219(68.0%)	1		
Yes	498(77.0%)	1.57(1.17–2.12)		0.003
**Had HIV-positive sexual partners**				
No	629(73.2%)	1		
Yes	88(80.0%)	1.46(0.90–2.39)		0.129
**Type of sexual partners**				
regular sexual partners	247(73.3%)	1		
casual sexual partners	470(74.4%)	1.06(0.78–1.43)		0.717
**Had sex with sex workers**				
No	680(74.2%)	1		
Yes	27(69.8%)	0.80(0.44–1.47)		0.476
**Had sex with female**				
No	638(74.5%)	1		
Yes	79(69.9%)	0.79(0.52–1.22)		0.293
**Consistent condom use**				
No	341(72.7%)	1		
Yes	376(75.2%)	1.14(0.85–1.52)		0.377
**Ever used rush popper**				
No	380(69.1%)	1		
Yes	337(80.4%)	1.84(1.36–2.49)		<0.001
**Heard of PrEP**				
No	220(67.3%)	1		
Yes	497(77.4%)	1.67(1.24–2.24)		0.001
**Willingness to use daily oral PrEP**				
No	85(37.4%)	1		
Yes	632(85.2%)	9.60(6.86–13.44)		<0.001
**Heard of PEP**				
No	234(67.2%)	1		
Yes	483(77.8%)	1.71(1.27–2.29)		<0.001
**Ever used PEP**				
No	650(73.2%)	1		
Yes	67(82.7%)	1.75(0.97–3.18)		0.065

### Multivariable factors associated with willingness to use LAI-PrEP

**Fig 1:**
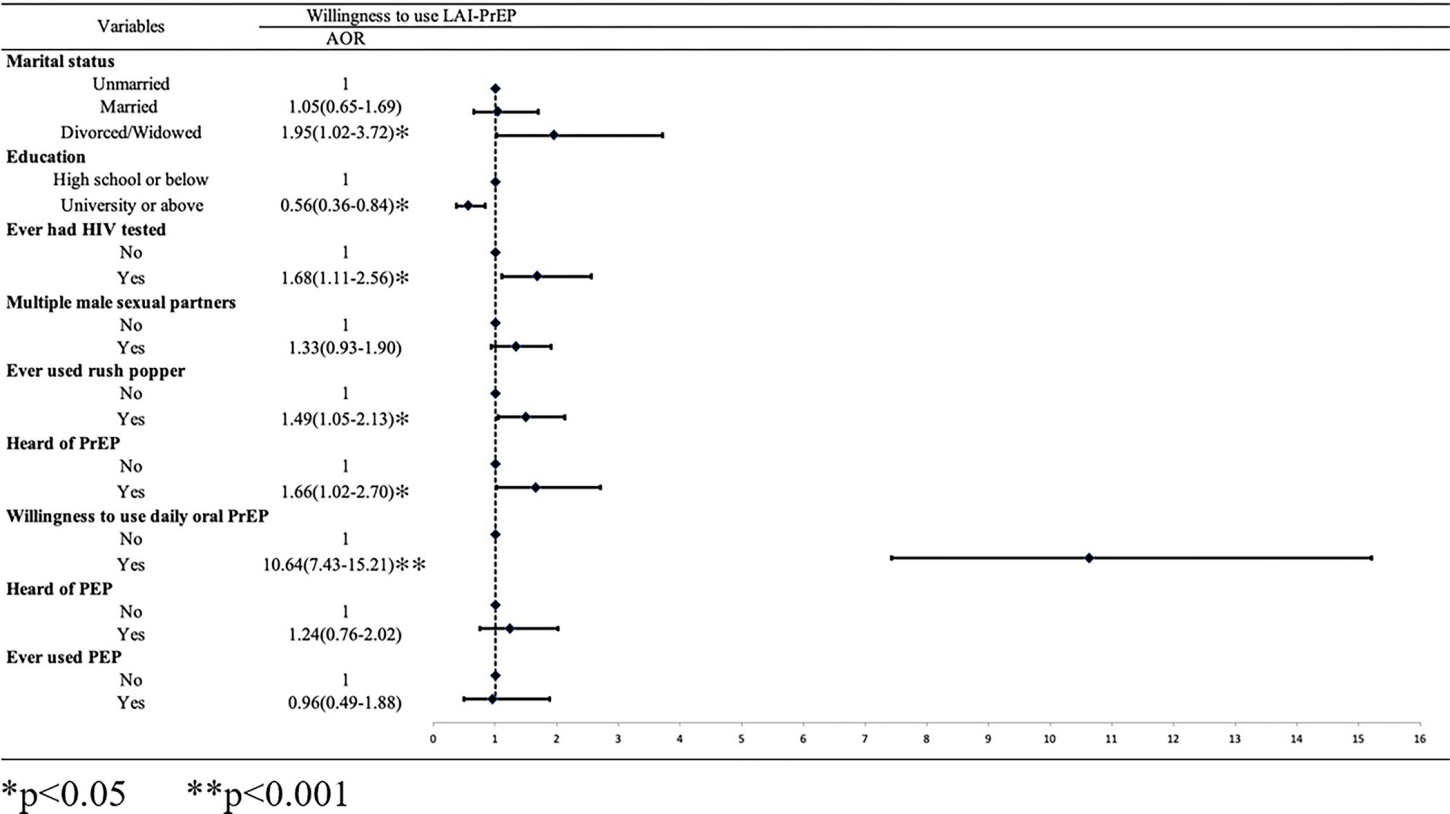
Multivariable factors associated with willingness to use LAI-PrEP.

In the multivariable model, MSM with higher education levels (AOR = 0.56, 95%CI: 0.38–0.84) were less likely to show a willingness to use LAI-PrEP. And participants with a history of HIV tests in their lifetime (AOR = 1.68, 95%CI: 1.11–2.56), had multiple male sexual partners (AOR = 1.33, 95%CI:0.93–1.90), ever used rush popper (AOR = 1.49, 95%CI:1.05–2.13) and ever hearing of PEP (AOR = 1.66, 95%CI: 1.02–2.70) were more likely to show willingness to use LAI-PrEP. Of note, MSM who were willing to use daily oral PrEP were more likely to use LAI-PrEP (AOR = 10.64,95%CI:7.43–15.21) (Fig 1).

### Reasons for unwillingness and willingness to use LAI-PrEP

**Fig 2:**
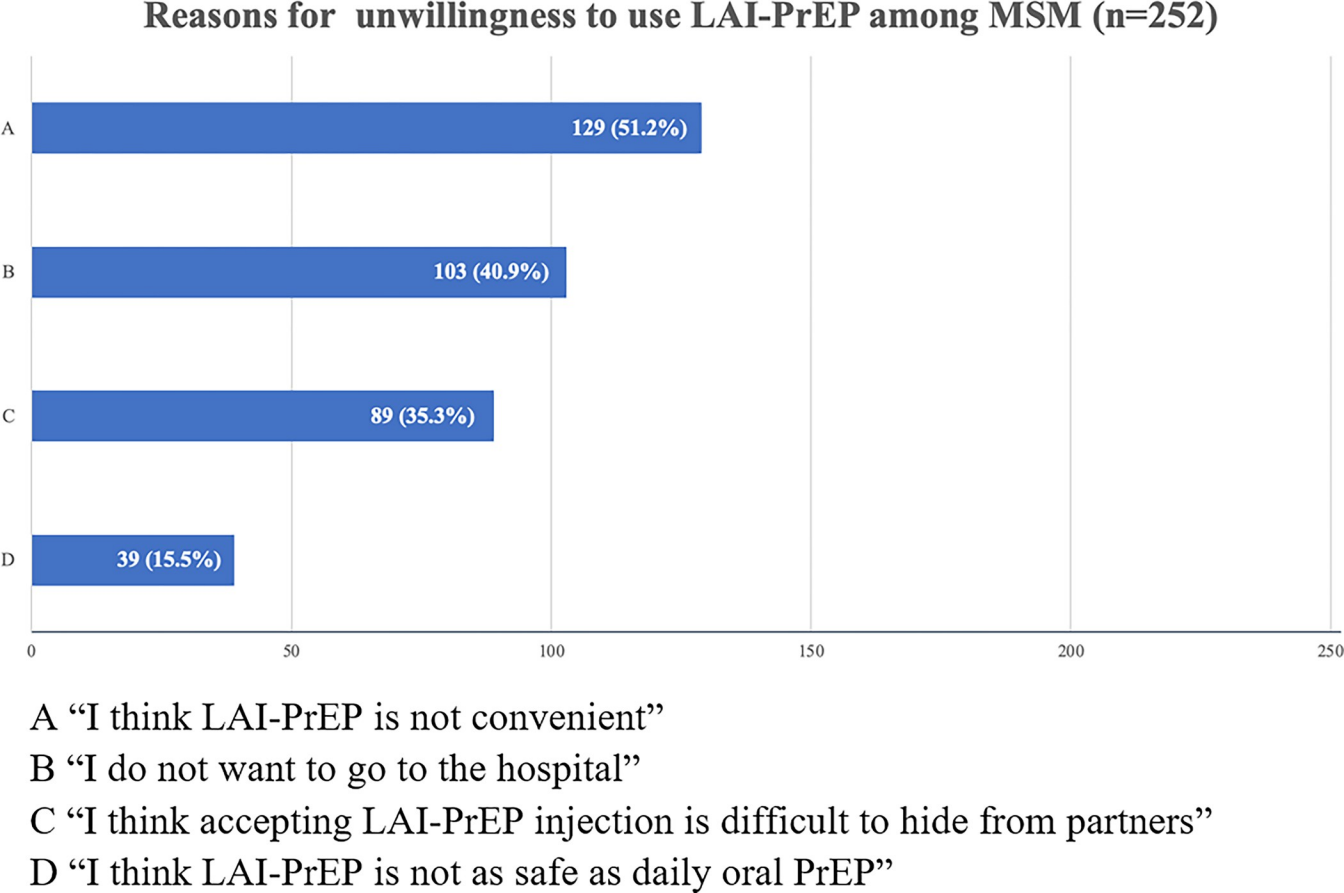
Reasons for unwillingness to use LAI-PrEP among MSM. A “I think LAI-PrEP is not convenient”. B “I do not want to go to the hospital”. C “I think accepting LAI-PrEP injection is difficult to hide from partners”. D “I think LAI-PrEP is not as safe as daily oral PrEP”.

**Fig 3:**
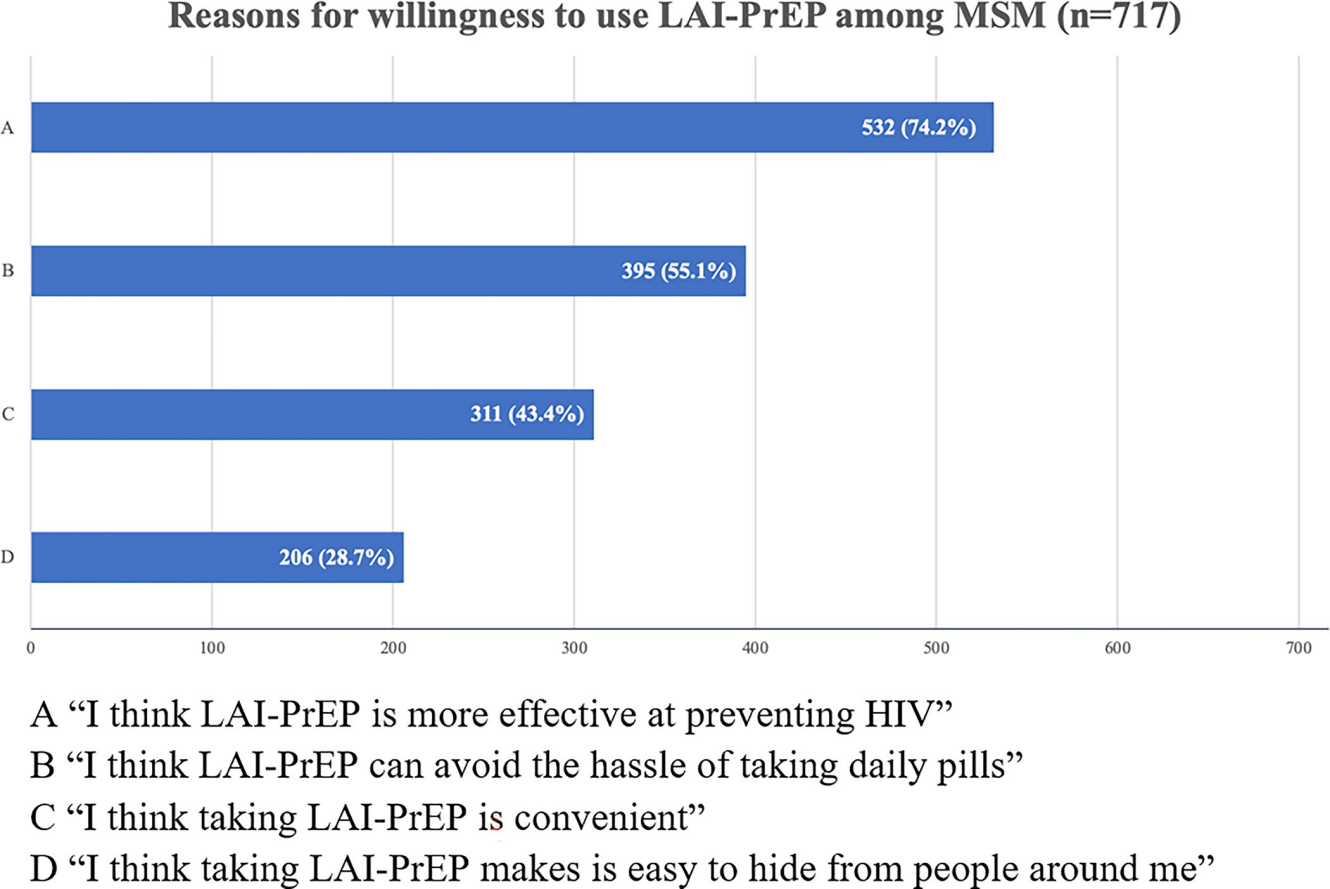
Reasons for willingness to use LAI-PrEP among MSM. A “I think LAI-PrEP is more effective at preventing HIV”. B “I think LAI-PrEP can avoid the hassle of taking daily pills”. C “I think taking LAI-PrEP is convenient”. D “I think taking LAI-PrEP makes is easy to hide from people around me”.

Among participants who were unwilling to use LAI-PrEP, most (51.2%) thought the daily oral PrEP was more convenient compared with LAI-PrEP. “Do not want to go to the hospital” (40.9%) and “LAI-PrEP was difficult to hide from partners” (35.3%) were also the main concerns for participants who were unwilling to use LAI-PrEP. Only 39 participants thought LAI-PrEP was not as safe as daily oral PrEP (Fig 2) Among participants who were willing to use LAI-PrEP, more than half thought LAI-PrEP was effective (74.2%) and decreased the trouble of daily pills (55.1%) (Fig 3).

## Discussion

This study investigated the willingness to use LAI-PrEP among MSM who were HIV-negative or serostatus-unknown in mainland China using a reliable and valid questionnaire. This is the first nationwide online study to explore the willingness to use LAI-PrEP among the Chinese MSM population.

The prevalence of consistent condom use (51.6%) among MSM in our study was higher than previous studies conducted in Shanghai (44.9%) and Chengdu (44.0%), but far lower than UNAIDS’ target (95%) [54–56]. In addition, we found that about two-fifths of MSM in our study used rush popper, one of the psychoactive substances, which could cause high-risk sexual behaviors and further increase the risk of HIV infection [57]. Previous research has identified various strategies for MSM to safeguard themselves against HIV and other sexually transmitted diseases, such as condom use and regular HIV testing, limiting the number of sex partners, and the use of dating apps [58,59]. Additionally, internet-based partner notification services and health services are available for HIV-negative MSM to protect against HIV and other STDs [60]. Apart from the preventive measures for reducing high-risk behaviors, it is important to seek additional efficacious alternatives to prevent HIV infection. PrEP, as an effective HIV prevention intervention, has been recommended by the WHO to prevent HIV infection [40]. It is necessary to explore some methods to increase the coverage of PrPE among MSM.

Overall, the results demonstrated that MSM had quite high awareness of PrEP (66.3%) and low uptake of PrEP (3.9%). The awareness of PrEP in this study is much higher than in other studies in mainland China, which ranged from 11.20% to 43.1% [1,15,61–63]. This could be because participants in our study were recruited via a gay-friendly app that routinely disseminates HIV knowledge to users. Spreading health information through apps that MSM use frequently may be an effective way to promote awareness of PrEP [64]. However, in most cases, the perceived willingness is not translated completely into actual action. The actual uptake of PrEP was only 3.9% in our study, which was slightly higher than previous research in Shanghai (2.5%), but far below the rate of uptake in the U.S. (60.5%) [42,65]. The gap between intention and behavior is common [66]. The high cost of PrEP is one of the reasons for its low uptake. In mainland China, for example, the cost of daily oral PrEP is around CNY 780–2000 (USD 111–285) per month. Another reason for poor uptake is concern about adverse effects of PrEP such as nausea, vomiting, and abdominal pain [67]. Someone concerned about adverse effects is unlikely to take action to use PrEP, since like all medications, PrEP may cause side effects. Common short-term effects include headaches, nausea, and stomach pain, which typically subside over time. Long-term use of PrEP pills has been associated with liver and kidney problems, as well as decreased bone density. Additionally, a previous study conducted in eastern China found that healthcare workers chose side effects as the main reason for being unwilling to accept tuberculosis prevention treatment [68]. Furthermore, due to traditional cultural beliefs about medicine, it is widely acknowledged in China that all drugs have some toxicity and that individuals are unwilling to take medicine until they have symptoms, which also acts as a roadblock to PrEP uptake among MSM at high risk of HIV infection [69]. It is urgent to explore a more effective strategy to narrow the gap between actual uptake of PrEP and perceived willingness to use PrEP. The transmission of HIV-related information, particularly through internet media, financial support, and destigmatizing PrEP and PrEP users are some strategies that have been suggested to bridge the gap between willingness and PrEP uptake behavior [70,71].

Compared to the studies conducted in Chengdu in 2018 (62.8%) and in other three southern cities (Guangzhou, Shenzhen, and Wuxi) in 2019 (38.5%), LAI-PrEP is more attractive for MSM in our study [1,40]. A possible explanation is that the above research was conducted earlier than the current study. Therefore, the coverage of HIV-related knowledge has been gradually expanding in recent years, which may lead to an increase in awareness of LAI-PrEP and willingness to use LAI-PrEP. Furthermore, unlike the previous recruiting method, our participants were recruited through a popular and widely used gay dating app. Previous studies proved that gay-dating app users had more sexual partners than non-app users [72]. This difference in sexual partners might be another reason why participants were more positive about PrEP usage in our study, as the medication would be more useful to these MSM than to those who have less frequent sex [73]. In accordance with Chen’s study, compared with participants recruited from sexual health clinics and gay-friendly health consulting service centers, MSM recruited from a gay dating app were more likely to be willing to use PrEP [1]. Thus, Lauren et al. pointed out that using gay-dating apps to deliver services or HIV prevention information is both cost-effective and potentially successful [72].

Consistent with prior research, our study found no association between income and willingness to use LAI-PrEP. This may be attributed to the fact that, despite potential concerns regarding cost, a substantial proportion of MSM expressed interest in utilizing LAI-PrEP due to its efficacy in preventing HIV infection [74]. However, a better educational background was found to be negatively associated with the willingness to use LAI-PrEP in our study. This result contradicts previous studies showing that MSM with higher education levels had a higher willingness to use PrEP [61,75,76]. When our survey was conducted, LAI-PrEP was still under clinical trial and had not been approved both in China and other countries. Participants were unfamiliar with this new product since they were unaware of its efficacy, side effects, and safety. Similar to the preventive vaccines that were not developed at the beginning of the COVID-19 pandemic, Metin et al. found that vaccine rejection increased significantly as education level increased [77]. Opel et al.’s research has also demonstrated that parents with higher education levels are approximately four times more likely to worry about vaccine safety than those with lower education levels [78]. Hence, MSM with higher education levels may be more cautious about the usage of LAI-PrEP because they do not know about all the benefits and drawbacks of this new PrEP and would show more hesitancy about LAI-PrEP that is still being tested. Participants who had multiple male sexual partners in the preceding 6 months had a higher likelihood of choosing LAI-PrEP, which was similar to a prior study finding that young MSM with a greater number of recent sexual partners were more likely to be willing to use LAI-PrEP [39]. One study conducted in Chengdu revealed that multiple male sexual partnerships increased the risk of contracting HIV [55]. Therefore, MSM were more likely to select LAI-PrEP for self-protection purposes when they perceived high risk for HIV acquisition [79]. When compared to individuals who were unwilling to use daily oral PrEP, those who were willing to use it were 10 times more likely to choose LAI-PrEP. This is in line with a previous study in a cohort that found that 95.4% of participants were interested in using LAI-PrEP among those who were willing to use daily oral PrEP [13]. It is possible that the individuals who were willing to use daily oral PrEP learned more about the significance of PrEP in HIV prevention and were more conscious of the challenges associated with daily medication. Thus, when participants learned that LAI-PrEP required less frequency and had the same effect as daily oral PrEP, they showed greater interest in LAI-PrEP [80].

About a third of participants in this study were unwilling to use LAI-PrEP, as an alternative formulation of daily oral PrEP. Here are some reasons. Firstly, individuals who are ready to use LAI-PrEP need to accept the injection at regular intervals. In addition to going to the hospital to accept injection, MSM who accept the LAI-PrEP need to take frequent blood tests to monitor the hepatotoxicity of injection [80]. Therefore, a series of inconveniences caused by receiving LAI-PrEP injections also hindered the willingness to choose PrEP among participants. Thirdly, MSM were also afraid of being found out about their HIV-related biomedical prevention measures [81]. In other words, receiving an HIV-related injection in the hospital will potentially lead to MSM being labeled as HIV-positive [80].

Our study also has some limitations. Firstly, sexual risk behaviors were self-reported. Participants might underreport such behaviors owing to social stigma. Therefore, the situation like multiple male sexual partners may be underestimated. Secondly, participants were recruited from a gay social networking app, whose users were younger and more educated than non-users [82]. The new information about HIV prevention may be easier to be accepted among this population. Therefore, the results from our study should be generalized to other MSM populations with caution. Thirdly, we did not consider those who started the survey but did not finish it, which may cause non-response bias. Fourthly, we did not investigate the awareness and preference for different types of PrEP among MSM. Hence, we could not further explore the preference for different modalities and associated influential factors. Fifthly, participants were not provided with information on the efficacy, side effects, cost, and regulatory approval of LAI-PrEP considering the length of the questionnaire and the main objectives of this study. Participants’ willingness to use LAI-PrEP may also be influenced by the above factors. Sixthly, this study is not a rigorous multicenter study, which could restrict the generalization ability of our results. However, this study covers most provinces in China, and could provide valuable insights into the willingness of the Chinese MSM population to receive LA-PrEP by identifying potential barriers and facilitators prior to its approval. These findings can inform the development of targeted policies and programs to promote the uptake of LA-PrEP following its approval.

## Conclusion

In our study, MSM had quite high awareness but low uptake of PrEP. As LAI-PrEP is expected to be approved for use in China in the future, our study of MSM highlights the need for key population-focused education programs about PrEP and healthy sexual behavior. This study also provides some evidence for LAI-PrEP use among the Chinese MSM population in the future.

## Supporting information

S1 Checklist STROBE statement—checklist of items that should be included in reports of observational studies. (DOCX)

S1 File Checklist for Reporting Results of Internet E-Surveys (CHERRIES). (DOCX)

S2 File (PDF)

S3 File (XLSX)

S4 File (DOCX)

S5 File Residence of participants. (DOCX)

## Abbreviations

MSM: Men who have sex with men
PrEP: Pre-exposure prophylaxis
LAI-PrEP: Long-acting injectable PrEP
PEP: Post-exposure prophylaxis
WHO: World Health Organization
UNAIDS: The Joint United Nations Programme on HIV/AIDS
