# Supplementary material for: Willingness to use long-acting injectable PrEP among HIV-negative/unknown men who have sex with men in mainland China: A cross-sectional online survey
Source: PLoS One. 2023 Oct 19;18(10):e0293297. doi: 10.1371/journal.pone.0293297 (PMC10586652; doi:10.1371/journal.pone.0293297)
Supplement: S2 File — (PDF) [file pone.0293297.s003.pdf]

淡蓝（北京）传媒有限公司
Danlan Beijing Media Limited

Suite 028, Block B, No.2, North of Apple Community,
Baiziwan Road, Chaoyang District, Beijing, China.
Tel: +86-10-58769855 Fax: +86-10-58769662

INSTITUTION REVIEW BOARD APPROVE LETTER

Grant #:

PROJECT TITLE: HIV intervention via geosocial-networking app among MSM

PRINCIPAL INVESTIGATOR: Xiaoyou Su, PhD

FUNDING AGENCY: Asian Regional Cooperation Fund, National Health Commission of PRC

DATE FOR WHICH REVIEWED: May 18, 2020

DATE APPROVED: May 20, 2020

DATE EXPIRED: April 30, 2021

The INSTITUTIONAL REVIEW BOARD of Danlan Beijing Media Limited (IRB00012406), has reviewed the proposed use of human subjects in the above-mentioned project. The right and the welfare of the subject are adequately protected; the potential risks are outweighed by potential benefits. No annual IRB review is required for this project, as permitted under the 2018 Common Rule.

Our IRB is registered with the U.S. Office for Human Research Protections and has a Federal Assurance FWA00029172.

Signature: Hongyan Lu

Lu Hongyan, M.D., Ph.D
Chair, Institutional Review Board of Danlan Beijing Media Limited
Date: May 20, 2020

淡蓝（北京）传媒有限公司伦理委员会
项目评审报告

项目编号：

经淡蓝（北京）传媒有限公司伦理委员会专家评审后，认为下列项目符合我国伦理有关要求，项目可以实施。

项目名称：基于网络开展男男性行为人群的健康干预研究

项目负责人：苏小游

单　　位：北京协和医学院公共卫生学院

评审日期：　2020 年 5 月 18 日

批准日期：　2020 年 5 月 20 日

有 效 期：　截止 2021 年 4 月 30 日

主席(签字)：

卢红艳

淡蓝（北京）传媒有限公司伦理委员会
