# Supplementary material for: Willingness to use long-acting injectable PrEP among HIV-negative/unknown men who have sex with men in mainland China: A cross-sectional online survey
Source: PLoS One. 2023 Oct 19;18(10):e0293297. doi: 10.1371/journal.pone.0293297 (PMC10586652; doi:10.1371/journal.pone.0293297)
Supplement: S4 File — (DOCX) [file pone.0293297.s005.docx]

**Cognitive and behavioral questionnaire for AIDS prevention and control**

1. If you agree to participate in this survey, please check the "Agree" option; If you do not agree, please check the "Disagree" option and your interests will not be affected in any way:

○Agree ○Disagree（Skip to the end and submit the questionnaire）

2. What is your gender?

○Male ○Female（Skip to the end and submit the questionnaire）

3. Are you over 18 years old?？

○Yes ○No（Skip to the end and submit the questionnaire）

**1. Sociodemographic information**

| 4. Your date of birth: ________year______month |
| --- |
| 5. Your nationality:  ○Han ○Other |
| 6. The city you currently live in：_______ |
| 7. Your marital status:  ○Single or unmarried ○married ○Marital separation ○divorced ○widowed |
| 8. Your current work/study situation:  ○Full-time employment (including freelance work) ○part-time jobs ○student ○retirement ○unemployed ○others(Please specify) |
| 9. Your education level:  ○Primary and below ○junior high school ○senior high school ○junior college ○bachelor degree ○Graduate students (including master's and doctoral students) |
| 10. Your average monthly income in the last 1 year is:  ○Less than 3000 ○3000-6999 ○7000-9999 ○10000-19999 ○20,000 and above |
| 11. What do you think your sexual orientation is:  ○homosexual ○bisexual ○heterosexuality ○indetermination ○others(Please specify) |
| 12. Have you ever been tested for HIV  ○Yes ○No（Jump to 16） |
| 13. When you were last tested for HIV:  ○Within three months ○4-6 months ago ○7-12 months ago ○More than 1 year |
| 14. The detection method is:  ○self-test ○center for disease control and prevention ○ hospital  ○community organization ○Others (please specify) |
| 15. The test results were:  ○ negative ○ positive |

**2. Sexual Behavior Characteristics**

16. Have you had anal sex with a man in the last 6 months?

○ Yes

○ No（Jump to 24）

17. How many men have you had anal sex with in the last 6 months? ______

18. How many of your male partners in the last 6 months have been HIV positive to your knowledge?

19. What type of male partners have you had in the last 6 months?

○Permanent partner (please fill in the number)______

○casual partners (please fill in the number)______

20. Permanent Partner (please fill in) : In the last 6 months, when you had anal sex with a male permanent partner, did you use a condom?

○ never

○ sometimes

○ always

21. Casual partner (please fill in) : When you have anal sex with a male permanent partner in the last 6 months, have you used a condom?

○ never

○ sometimes

○ always

22. Have you had commercial sex in the last 6 months?

○ Yes

○ No

23. Have you used a condom during commercial sex in the last 6 months? (Only displayed when question 22 selects "yes")

○ Never

○ Sometimes

○ Always

24. Have you had sex with a woman in the last 6 months?

○ Yes

○ No

25. In the last 6 months, have you used a condom when having sex with a woman? (Only displayed when question 24 selects "yes")

○ Never

○ Sometimes

○ Always

26. What is the reason you have not used a condom during sex with your partner in the last 6 months? (Multiple choice)

○Always use a sleeve (mutually exclusive with other options)

○Condoms do not reduce the risk of HIV infection

○The other half doesn't want to use

○I forget when I am in a hurry

○Thinking "I" can't get HIV

○Not available near the premises

○The condom accidentally breaks or slips

○Know the protection of condoms, but during sex, condoms accidentally slip off

○Other reasons (please specify)

27. Have you ever used meth in the last six months?

○Yes ○No

28. Have you used rush (poppers) in the last 6 months?

○Yes ○No

29. How many men have you had anal sex with in the last 6 months?

○0-5 ○6-10 ○11-15 ○16 and above

**3. HIV-related knowledge——HIV-KQ-18**

| **Items** | **True** | **False** | **Unknown** |
| --- | --- | --- | --- |
| 30、Coughing and sneezing do not spread HIV.（T） |  |  |  |
| 30、You can contract HIV by sharing a glass of water with an HIV-infected person.（F） |  |  |  |
| 30、Men pulling out their penis before reaching orgasm can prevent women from contracting HIV during sex.（F） |  |  |  |
| 30、A woman can get HIV by having anal sex with a man. （T） |  |  |  |
| 30、After having sex, you can prevent HIV infection by showering or washing your genitals/private parts.（F） |  |  |  |
| 30、Every pregnant woman infected with HIV will give birth to a child with AIDS.（F） |  |  |  |
| 30、People infected with HIV quickly show serious signs of being infected.（F） |  |  |  |
| 30、There is a vaccine that can prevent adults from contracting HIV.（F） |  |  |  |
| 30、If your partner is infected with HIV, people are likely to contract HIV through deep kissing - putting their tongue in their partner's mouth.（F） |  |  |  |
| 30、Women who have sex during menstruation cannot get HIV.（F） |  |  |  |
| 30、There is a female condom that can help reduce a woman's chances of contracting HIV.（T） |  |  |  |
| 30、Natural skin condoms provide better protection against HIV than latex condoms.（F） |  |  |  |
| 30、People who are taking antibiotics will not contract HIV.（F） |  |  |  |
| 30、Having sex with multiple partners can increase your chances of contracting HIV.（T） |  |  |  |
| 30、An HIV test one week after having sex can determine whether you are infected with HIV.（F） |  |  |  |
| 30、You can get HIV in the same hot tub or swimming pool as someone with HIV.（F） |  |  |  |
| 30、You can get HIV from oral sex.（T） |  |  |  |
| 30、Using Vaseline or baby oil with a condom can reduce the chance of contracting HIV.（F） |  |  |  |

**4. depression（CES-D_10_）**

Below are 10 statements. Choose the one that best matches your feelings about the past week.

|  | No or rarely (less than 1 day) | Sometimes or a small amount of time (1-2 days) | Often or half the time (3-4 days) | Most or all of the time (5-7 days) |
| --- | --- | --- | --- | --- |
| 31、I feel blue. | 0 | 1 | 2 | 3 |
| 31、I find it difficult to do anything. | 0 | 1 | 2 | 3 |
| 31、I don't sleep well. | 0 | 1 | 2 | 3 |
| 31、I feel happy. | 0 | 1 | 2 | 3 |
| 31、I feel lonely. | 0 | 1 | 2 | 3 |
| 31、I don't think people are very friendly to me. | 0 | 1 | 2 | 3 |
| 31、I find life interesting. | 0 | 1 | 2 | 3 |
| 31、I feel sad. | 0 | 1 | 2 | 3 |
| 31、I don't think people like me. | 0 | 1 | 2 | 3 |
| 31、have no motivation to work. | 0 | 1 | 2 | 3 |

**5. Awareness of HIV prevention drugs (11 questions) (only HIV-negative or untested people answered)**

| 32、Have you ever heard of pre-exposure prophylaxis (PrEP)?  ○Yes  ○No（Jump to 35） |
| --- |
| 33、As far as you know, if used correctly, the effectiveness of pre-exposure drugs for HIV prevention is:  ○30% and above  ○50% and above  ○70% and above  ○90% and above  ○I don’t know |
| 34、Have you ever used pre-exposure prophylaxis (PrEP)?  ○Yes（Jump to 37） ○No |
| 35、If taking pre-exposure prophylaxis (PrEP) every day or continuously before and after sex is effective in preventing HIV infection, would you consider using prep in the future?  ○Quite willing（Jump to 37）  ○willingness（Jump to 37）  ○unknown  ○unwillingness  ○Completely unwilling |
| 36、What is the reason for your reluctance to use it? (Multiple choice)  ○There is no sexual activity or sexual partner fixation  ○Reluctant to take medicine for fear of side effects  ○No high-risk sexual behavior  ○Would rather go with the PEP  ○Privacy concerns would expose me as a gay man  ○Not willing to spend extra money  ○Others (please specify) |
| 37、Would you prefer to take a long-acting PrEP every two months rather than a daily oral pre-exposure prophylaxis pill?  ○Quite willing（Jump to 39）  ○willingness（Jump to 39）  ○unknown  ○unwillingness  ○Completely unwilling |
| 38、What's the reason you don't want to take the shot? [Multiple choice]  ○Convenient for oral medicine  ○Oral medicine is safe and its efficacy is guaranteed  ○No need to go to the hospital  ○Oral medicine is not easy to detect  ○Others (please specify) |
| 39、Why are you willing to take the injection? [Multiple choice]  ○Avoid the hassle of taking daily pills  ○Convenient, will not forget  ○Not easy for anyone to see  ○Can be more effective in preventing HIV  ○Others (please specify) |
| 40、Have you ever heard of post-exposure prophylaxis (PEP) before?  ○Yes ○No |
| 41、To your knowledge, post-exposure prophylaxis (PEP) should be started within how many hours of high-risk behavior? (Who has heard of PEP in only 40 questions?)  ○24 hours ○36 hours ○48 hours ○72 hours |
| 42、Have you ever used post-exposure prophylaxis (PEP)? (Who has heard of PEP in only 40 questions?)  ○Yes ○No |

**6. HIV anticipated Stigma Scale**

|  | strongly disagree | disagree | agree | Totally agree |
| --- | --- | --- | --- | --- |
| 43、1. If I were HIV-positive, I would worry about being discriminated against. | □ | □ | □ | □ |
| 43、2. If I were HIV-positive, no one would want to be in a relationship or date me. | □ | □ | □ | □ |
| 43、3. If I were HIV-positive, no one would want to have sex with me. | □ | □ | □ | □ |
| 43、4. If I were HIV-positive, I would try to keep it a secret. | □ | □ | □ | □ |
| 43、5. If I knew I had HIV, I would feel isolated from the rest of the world. | □ | □ | □ | □ |
| 43、6. If I were living with HIV, I would feel inferior. | □ | □ | □ | □ |
| 43、7. I would never be ashamed to have HIV. | □ | □ | □ | □ |

**7. IMB model**

| **AIDS related awareness** | | | | | |
| --- | --- | --- | --- | --- | --- |
|  | strongly disagree | Disagree | Not sure | Agree | Totally agree |
| 44、1. The thought of getting AIDS scares me | □ | □ | □ | □ | □ |
| 44、2. HIV infection is very serious and affects my daily life | □ | □ | □ | □ | □ |
| 44、3. Having AIDS was devastating for me | □ | □ | □ | □ | □ |
| 44、4. I think I'm at risk of getting AIDS | □ | □ | □ | □ | □ |
| 44、5. Consistent condom use helps protect me from HIV infection | □ | □ | □ | □ | □ |
| 44、6. Using PrEP/PEP helped protect me from HIV infection | □ | □ | □ | □ | □ |
| 44、7. Regular testing can help you know your infection status and seek help | □ | □ | □ | □ | □ |
| 44、8. Not using AIDS like rush reduces my risk of HIV infection | □ | □ | □ | □ | □ |
| 44、9. I doubt the safety and effectiveness of PrEP/PEP | □ | □ | □ | □ | □ |
| 44、10. It's hard for me to decide whether to stick to condoms | □ | □ | □ | □ | □ |
| 44、11. I'm having a hard time deciding whether to stick with rush or not | □ | □ | □ | □ | □ |
| **subjective norm** | | | | | |
|  | Strongly disagree | disagree | Not sure | agree | Totally agree |
| 45、1. Most significant people around me believe that I should discuss safe sex with my partners before having sex | □ | □ | □ | □ | □ |
| 45、2 Most significant people around me believe that I should try to persuade my partner to only have safe sex before having sex | □ | □ | □ | □ | □ |
| 45、3. Most significant people around me believe that I should buy or have condoms ready before having sex | □ | □ | □ | □ | □ |
| 45、4. Most significant people around me believe that I should insist on using a condom when having sex | □ | □ | □ | □ | □ |
| 45、5. Most important people around me think I should get tested regularly for HIV | □ | □ | □ | □ | □ |
| 45、6.. Most of the important people around me think I shouldn't abuse sex AIDS | □ | □ | □ | □ | □ |
| 45、7. In general, I do what the important people around me think I should do | □ | □ | □ | □ | □ |
| **Behavior skill** | | | | | |
|  | Strongly disagree | disagree | Not sure | Agree | Totally agree |
| 46、1. I can decide for myself whether to take AIDS protection or not | □ | □ | □ | □ | □ |
| 46、2. I take my health very seriously | □ | □ | □ | □ | □ |
| 46、3. Preventing disease and infection is important to me | □ | □ | □ | □ | □ |
| 46、4. I'm happy to discuss HIV prevention with potential partners | □ | □ | □ | □ | □ |
| 46、5. I have the confidence to refuse to have sex with a man who is not willing to use AIDS protection | □ | □ | □ | □ | □ |
| 46、6. I am confident that I can persuade my sexual partners to take AIDS protection measures | □ | □ | □ | □ | □ |
| 46、7. I can take AIDS protection if I want to | □ | □ | □ | □ | □ |
| 46、8. I was willing to get tested for HIV even though I was afraid of knowing the result | □ | □ | □ | □ | □ |
| 46、9. I can get tested regularly for HIV | □ | □ | □ | □ | □ |
| 46、10. I know how to take AIDS protection measures to protect myself | □ | □ | □ | □ | □ |

**9. The problem of ART compliance rate of infected persons**

| 47、Are you receiving antiviral treatment?  （1）Yes （Jump to 49） （2）No |
| --- |
| 48、What's the reason you're not taking antiviral therapy? [Multiple options available]  （1）I feel that I am in good health and do not want treatment for the time being  （2）Afraid to reveal himself as infected  （3）Losing faith in life, giving up therapy  （4）Fear of drug side effects  （5）Financial difficulties, unwilling to bear the cost of testing  （6）It's not convenient to run back and forth in the field for a long time  （7）Other diseases or complications need to be controlled first  （8）others |
| 49、How long have you been taking antiviral drugs  （1）1-3 months  （2）4-6 months  （3）7-12 months  （4）1-2 years  （5）2years above |
| 50、In what way do you remind yourself to take your medication? [Multiple options available]  （1）Family reminder  （2）Alarm clock reminder  （3）Message reminder  （4）Calendar record  （5）Others  （6）None |
| 1. In the last six months, have you ever missed taking medicine?   （1）Yes （2）No（Jump to 53） |
| 52、Why you missed your medication?  （1）I don't think I need any medicine  （2）Forget to take medicine  （3）Take too many pills  （4）There is no medicine.  （5）too busy  （6）It's not convenient to get the medicine  （7）Missed medication  （8）The drug had an adverse reaction  （9）Go out without medicine  （10）Complicating other diseases  （11）I don't want anyone to know I'm on my meds  （12）Others |
| 53、Have you ever heard of long-acting AIDS injectable drugs that are given once a month or once every two months?  （1）Yes （2）No |
| 54、Would you be willing to take a long-acting injectable drug given monthly or bimonthly instead of an oral antivirals?  ○Quite willing  ○willingness  ○unknown  ○unwillingness  ○Completely unwilling |
| 55. For you, what are your biggest concerns about long-acting injectable drugs for AIDS? [Multiple choice]  （1）Drug effect  （2）side effect  （3）tolerance  （4）approach of achieving  （5）Price  （6）Without any worry  （7）Others |
| 56. What's the reason you don't want to take long-acting injectable drugs? [Multiple choice]  （1）Convenient for oral medicine  （2）Oral medicine is safe and its efficacy is guaranteed  （3）Oral medicine does not need to go to the hospital to inject  （4）Oral medicine is not easy to detect  （5）Oral medicine may be cheap  （6）Others |

1. **IMB module - Infected**

| **AIDS related awareness** | | | | | |
| --- | --- | --- | --- | --- | --- |
|  | Strongly disagree | Disagree | Not sure | Agree | Totally agree |
| 57、1. The thought of having AIDS terrifies me | □ | □ | □ | □ | □ |
| 57、2. The AIDS infection is very serious and affects my daily life | □ | □ | □ | □ | □ |
| 57、3. Having AIDS was devastating for me | □ | □ | □ | □ | □ |
| 57、4. Consistent use of antiviral drugs can help treat HIV infection | □ | □ | □ | □ | □ |
| 57、5. Regular testing can help you understand your condition and seek further help | □ | □ | □ | □ | □ |
| 57、6. I doubt the safety and effectiveness of antivirals | □ | □ | □ | □ | □ |
| 57、7. It's hard for me to decide whether to stick to antiviral drugs | □ | □ | □ | □ | □ |
| **subjective norm** | | | | | |
|  | Strongly disagree | Disagree | Not sure | Agree | Totally agree |
| 58、1. Most significant people around me believe that I should discuss safe sex with my partners before having sex | □ | □ | □ | □ | □ |
| 58、2. Most significant people around me believe that I should try to persuade my partner to only have safe sex before having sex | □ | □ | □ | □ | □ |
| 58、3. Most significant people around me believe that I should buy or have condoms ready before having sex | □ | □ | □ | □ | □ |
| 58、4. Most significant people around me believe that I should insist on using a condom when having sex | □ | □ | □ | □ | □ |
| 58、5. Most of the important people around me think that I should get regular medical check-ups | □ | □ | □ | □ | □ |
| 58、6. Most of the important people around me believe that I should remain compliant | □ | □ | □ | □ | □ |
| 58、7. In general, I do what the important people around me think I should do | □ | □ | □ | □ | □ |
| **Behavior skill** | | | | | |
|  | Strongly disagree | Disagree | Not sure | agree | Totally agree |
| 59、1. I can decide for myself whether to stick with the antiviral | □ | □ | □ | □ | □ |
| 59、2. I take my health very seriously | □ | □ | □ | □ | □ |
| 59、3. I am happy to discuss HIV/AIDS with potential partners | □ | □ | □ | □ | □ |
| 59、4. I have the confidence to refuse to have sex with a man who is not willing to use AIDS protection | □ | □ | □ | □ | □ |
| 59、5. I am confident that I can persuade my sexual partners to take AIDS prevention measures | □ | □ | □ | □ | □ |
| 59、6. I can get regular CD4 and viral load tests | □ | □ | □ | □ | □ |
| 59、7. I know how to take AIDS prevention measures | □ | □ | □ | □ | □ |

**11. HIV Stigma Scale**

|  | Much | Some | A lot | None |
| --- | --- | --- | --- | --- |
| 60、1. Because I have AIDS, I feel that the people around me are distant, no longer close to me | □ | □ | □ | □ |
| 60、2. Because I have AIDS, I feel that people around me are complaining and blaming me | □ | □ | □ | □ |
| 60、3. Our own family will be distant, do not care about the family of AIDS infected people | □ | □ | □ | □ |
| 60、4. Because I have AIDS, I feel that it is difficult for me or my family to get a wife or marry | □ | □ | □ | □ |
| 60、5. Because I have AIDS, I feel uncomfortable with the people around me | □ | □ | □ | □ |
| 60、6. Because of my AIDS, I felt that people could no longer see my good points | □ | □ | □ | □ |
| 60、7. Because I had AIDS, I felt that parents stopped letting their children near me | □ | □ | □ | □ |
| 60、8. Because I have AIDS, I feel that my family will not get the same right to education as others | □ | □ | □ | □ |
| 60、9. Because I have AIDS, I feel that it is not easy for me or my family to find a job | □ | □ | □ | □ |

**12．Suicidal ideation and suicide attempts/social support**

|  | Yes | No |
| --- | --- | --- |
| 61、1. Have you had suicidal thoughts in the last six months, even though you didn't do it? | □ | □ |
| 61、2. In the last six months, have you tried to kill yourself? | □ | □ |
| **Here are some questions about your social support. Please use a scale of 0-10, 0 being none at all and 10 being very much. (Please fill in integers)** | | |
| 62、How much support do you get from your family/friends/colleagues when you need to talk or emotional support? | | |
| 63、How much support can you get from your family/friends/colleagues when you need material help (e.g. financial hardship)? | | |
