# Supplementary material for: Willingness to use long-acting injectable PrEP among HIV-negative/unknown men who have sex with men in mainland China: A cross-sectional online survey
Source: PLoS One. 2023 Oct 19;18(10):e0293297. doi: 10.1371/journal.pone.0293297 (PMC10586652; doi:10.1371/journal.pone.0293297)
Supplement: S5 File — (DOCX) [file pone.0293297.s006.docx]

**Residence of participants**

|  | Frequency | Percentage(%) |
| --- | --- | --- |
| Anhui | 4 | 0.4 |
| Beijing | 26 | 2.7 |
| Fujian | 5 | 0.5 |
| Gansu | 1 | 0.1 |
| Guangdong | 93 | 9.6 |
| Guangxi | 159 | 16.4 |
| Guizhou | 6 | 0.6 |
| Hainan | 3 | 0.3 |
| Hebei | 124 | 12.8 |
| Henan | 2 | 0.2 |
| Heilongjiang | 3 | 0.3 |
| Hubei | 41 | 4.2 |
| Hunan | 25 | 2.6 |
| Jilin | 5 | 0.5 |
| Jiangsu | 5 | 0.5 |
| Jiangxi | 122 | 12.6 |
| Liaoning | 168 | 17.3 |
| Inner Mongolia | 5 | 0.5 |
| Shandong | 5 | 0.5 |
| Shanxi | 6 | 0.6 |
| Shaanxi | 9 | 0.9 |
| Shanghai | 9 | 0.9 |
| Sichuan | 9 | 0.9 |
| Tianjin | 8 | 0.8 |
| Xizang | 1 | 0.1 |
| Xinjiang | 11 | 1.1 |
| Yunnan | 7 | 0.7 |
| Zhejiang | 97 | 10.0 |
| Chongqing | 10 | 1.0 |
| Total | 969 | 100.0 |
